# Post Disaster Damage Assessment Using Ultra-High-Resolution Aerial Imagery with Semi-Supervised Transformers

**DOI:** 10.3390/s23198235

**Published:** 2023-10-03

**Authors:** Deepank Kumar Singh, Vedhus Hoskere

**Affiliations:** Department of Civil and Environmental Engineering, University of Houston, Houston, TX 77204, USA; dksingh@uh.edu

**Keywords:** preliminary damage assessments, transformers, semi-supervised learning, aerial imagery

## Abstract

Preliminary damage assessments (PDA) conducted in the aftermath of a disaster are a key first step in ensuring a resilient recovery. Conventional door-to-door inspection practices are time-consuming and may delay governmental resource allocation. A number of research efforts have proposed frameworks to automate PDA, typically relying on data sources from satellites, unmanned aerial vehicles, or ground vehicles, together with data processing using deep convolutional neural networks. However, before such frameworks can be adopted in practice, the accuracy and fidelity of predictions of damage level at the scale of an entire building must be comparable to human assessments. Towards this goal, we propose a PDA framework leveraging novel ultra-high-resolution aerial (UHRA) images combined with state-of-the-art transformer models to make multi-class damage predictions of entire buildings. We demonstrate that semi-supervised transformer models trained with vast amounts of unlabeled data are able to surpass the accuracy and generalization capabilities of state-of-the-art PDA frameworks. In our series of experiments, we aim to assess the impact of incorporating unlabeled data, as well as the use of different data sources and model architectures. By integrating UHRA images and semi-supervised transformer models, our results suggest that the framework can overcome the significant limitations of satellite imagery and traditional CNN models, leading to more accurate and efficient damage assessments.

## 1. Introduction

Preliminary damage assessments (PDA) evaluate the extent of damage caused by disasters to buildings and are the first step in the post-disaster recovery process [1,2]. These damage assessments are necessary after disasters to ensure the safety of buildings and allocate government resources to homeowners. The PDA process begins with an initial damage assessment (IDA) [3], where damage information is collected and verified by state or tribal authorities via door-to-door surveys over the affected regions. Individual assessments (IA) [4] are conducted as part of the initial damage assessment (IDA) [4] for each disaster-affected home. IAs are conducted door-to-door as disaster victims apply for aid but are inefficient and pose safety risks. For instance, after Hurricane Ian, victims had to wait nearly five months after the storm [5,6] to have their IAs completed. During inspections, compromised components and hazardous debris hamper the ability of inspectors to reach all areas safely and enter damaged properties [7,8,9,10,11]. A large disaster could potentially result in hundreds of thousands of IA applications, overwhelming the available workforce and rendering the number of inspectors and support staff inadequate to meet the demands of comprehensive evaluations [6,8]. There is thus a need for alternative methods that can help accelerate the PDA process.

A time-consuming step of the PDA process is the identification of the damage state of individual buildings. Researchers have proposed solutions to enable faster and safer post-disaster damage state assessments [8,9,10,11]. These solutions typically rely on one or more sources of data that can be obtained in an automated or efficient manner, combined with data processing methods that exploit computer vision and deep learning that can extract actionable information like the damage state of structures [12,13,14,15].

Different data sources have been studied for their suitability for post-disaster damage assessments including images captured via satellites (optical and SAR) [16,17,18,19,20,21,22,23,24], unmanned aerial vehicles (UAVs) [25,26,27,28,29], and ground-level cameras [30,31,32]. Satellite images are the most commonly utilized data source due to their wide availability for larger regions. For example, the xBD dataset offers an extensive compilation of pre- and post-event satellite imagery, building polygons annotated with four damage levels [21,22]. Among the numerous recent studies conducted on the xBD dataset, Bai et al. trained a model on satellite data and tested its generalizability on the 2011 Tohoku earthquake. Two major concerns with satellite imagery are the reduced visibility during overcast conditions and the limited resolution available that limit the accuracy of damage identification [33]. Other researchers have proposed methods that utilize both pre- and post-disaster satellite imagery for building damage assessment. However, there are instances where pre-disaster images may not be always available [34,35,36,37]. Additionally, synthetic aperture radar (SAR) images offer an alternative to optical satellite images, overcoming overcast limitations and enhancing satellite-based image analysis for various applications [17,18,19,20,21,38,39]. SAR images still pose a challenge for reliable individual building assessment due to their very low resolution. UAV data on the other hand provides high-resolution images compared to satellite data yielding higher quality assessments in comparison. Gerke et al. [40], and others [26,27,29] have utilized the EMS-98 classification system, which categorizes residential buildings into five damage classes. Through their study [40], the authors investigated the varied and uncertain nature of observed damage patterns in different damage classes [11]. Additionally, several studies have demonstrated the use of UAVs in automating post-earthquake assessments [31,32]. UAV data offers high-resolution images but has limitations like flight time, restricted coverage area, and weather dependency, impacting its utility for post-disaster assessments. Regarding the third data type, researchers have made use of ground-level camera images for post-disaster assessments [30,41,42]. These images offer a complementary close-up view’s perspective to satellite imagery for detailed assessment but are difficult to scale over larger regions and have accessibility and safety concerns. All these studies suggest that each datatype comes with its own set of limitations, further emphasizing the need for careful consideration when utilizing different sources to enable more efficient PDA.

In addition to visual data, researchers have utilized other dynamic data sources like wind speed, ground motion data like PGA (Peak Ground Acceleration), and response spectra [43]. A paper by Lombardo et al. presents an approach to the use of Monte Carlo simulation to quantify the misclassification of tornado characteristics by establishing a relationship between the degree of damage and wind speed [44]. Yuan et al. introduced a 1D CNN-based approach for damage assessment [45,46]. Moreover, ground motion data provides an advantage in assessing underground structural damage as discussed in studies [47,48,49].

In addition to the data source, the choice of post-processing methods to extract actionable information plays a crucial role in determining the accuracy of assessments. Researchers have explored various heuristic and deep learning methods for tasks such as damage classification and change detection algorithms. Most of the analysis with satellite images focuses on bitemporal images, which consist of pre- and post-disaster images. By utilizing bitemporal satellite images, it becomes possible to visually observe differences since disasters often lead to significant changes in the imagery. Several researchers have focused on detecting these changes by employing pixel-to-pixel comparison methods [24,50] as well as deep learning techniques [26,27,33,35,51]. In a case study of Hurricane Michael, Berezina et al. [10] utilized a U-Net model for segmentation and a ResNet CNN architecture for classification on the segmented images. The results demonstrated the clear superiority of deep neural network architectures like CNNs over the support vector machines classifier for change detection with satellite images. Similarly, Hong et. al [9] presented a novel network called EBDC-Net to solve the finer classification problem of damaged buildings after earthquakes. Many papers focusing on change detection algorithms are restricted by a limited number of damage classes, typically only two classes (binary classification problem) limiting the usable insight about the damage state of a building [9,30,34,52,53]. In a recent study by Khajwal et al. [30], a multi-class classification study using a dataset of around 500 post-disaster building images revealed an initial accuracy of approximately 55% when utilizing a single aerial image. Additionally, by incorporating multi-view images into their analysis, the authors achieved an additional 10% increase in accuracy.

While these advancements described above represent significant progress in the development of a dependable damage assessment tool, they still fall short of human-level performance at 70% [54] for satellite images and thus leave room for improvement. To advance the development of an automated PDA framework, it is crucial to thoroughly investigate novel data sources and methodologies in an integrated manner. There is an inherent tradeoff between using satellite imagery and images from UAVs. Satellite images lack the necessary level of detail required for accurate model predictions. UAV images on the other hand are difficult to acquire over large areas due to limited speeds, flight time, privacy concerns, and range. With regard to the computer vision methodologies utilized, recent advances that leverage unlabeled data typically available in quantities that are orders of magnitude larger than labeled images have received limited attention [55,56,57]. Additionally, existing research has predominantly employed convolutional neural network (CNN) models, while recent findings for other applications suggest that transformers may offer superior performance [58,59,60] and thus require investigation towards their applicability for PDA.

**Figure 1:**
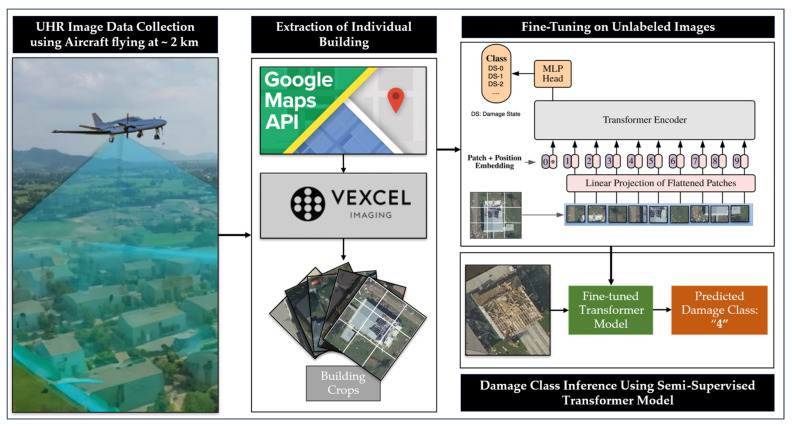
Proposed PDA Framework.

We propose a new framework (Figure 1) for PDA, leveraging novel ultra-high-resolution aerial (UHRA) imagery, together with semi-supervised learning techniques to utilize vast amounts of unlabeled data and enhance the consistency and accuracy of multi-class damage classification to surpass human levels. The novel contribution of our research comes from adopting (i) UHRA images, (ii) unlabeled data into the training pipeline, and (iii) vision-transformer models. We study the effect of the data type and compare our proposed processing method to state-of-the-art approaches to demonstrate the superior performance of our proposed framework over those state-of-the-art approaches. Section 2 outlines our data collection and preparation process, including UHRA and satellite image data, along with introducing the supervised vision transformer (ViT) and semi-supervised Semi-ViT models as part of our deep learning architectures. Section 3 comprises three key experiments: semi-supervised learning with unlabeled data, comparison of CNN and transformer model architectures, and comparison of satellite and UHRA image data types. In Section 4, we delve into the results of each experiment, analyzing their implications and significance. Finally, we conclude the paper in Section 5, summarizing our findings and limitations.

## 2. Proposed Methods

Our framework for PDA is illustrated in Figure 1. The process consists of four steps. Firstly, raw UHRA image data is collected using an aircraft equipped with an ultra-high-resolution image sensor, (e.g., UltraCam from Vexcel Imaging), typically within 2–3 days after a hurricane strikes. For instance, after Hurricane Michael, the data for an 85,000 km^2^ area across four states was published online in just over three days [61]. Then, the collected data is processed to extract individual building crops in an automated fashion. A pre-trained transformer model is then fine-tuned on the unlabeled building crops in an unsupervised manner to learn the distribution of the newly acquired data. Finally, the fine-tuned network is used to predict the damage class.

Our research methodology involved in developing the proposed framework examined different data sources and the deep learning architectures described in this section.

### 2.1. Data Sources, Collection, and Preparation

We compare the efficacy of images from two data sources: satellite images from Google satellite images [62,63] and UHRA images from Vexcel Imaging [64].

**Figure 2:**
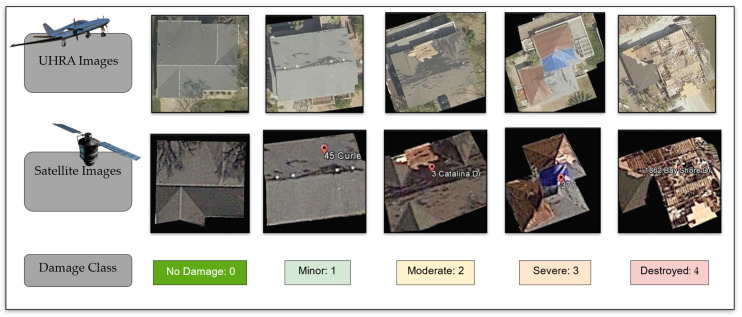
Samples for UHRA and satellite images with corresponding damage classes.

In this study, we use a 5-class scale for building damage, numbered 0 to 4, representing the severity of the damage. The ground truth is obtained from field observations by Kijewaski-Correa et al. [65] made available through NEHRI Design Safe [66]. A 5-class scale was chosen because it aligns with the visually identifiable classes for FEMA individual assessments (IA) [4] and the HAZUS resistance model [67]. The criteria used to define the damage classes have been discussed in [4,67]. The correspondence between the classes taken on in this study is provided in Table 1. Example UHRA and satellite images for each damage class in the proposed framework are provided together in Figure 2.

In the upcoming two sections, we will provide a detailed explanation of the extraction process for both types of images collected from different data sources.

#### 2.1.1. UHRA Image Data

The UHRA images used in this study were acquired from Vexcel Imaging [64]. The images are captured via a fleet of fixed-winged aircraft equipped with the UltraCam, a high-resolution camera system, to capture up to 1.7 cm ground sample distance (GSD), overcoming the limitation of SAR and satellite images (usually 30–50 cm GSD) [69]. Unlike arial images captured using drones, UHRA images can also be quickly acquired by aircraft over a large area in a short span of time [61]. Furthermore, UHRA images mitigate the constraints associated with ground images, as they do not pose accessibility issues or safety concerns.

Our dataset was built using multiple online resources, including DesignSafe [66], the Google Maps Geocoding API [70], and Vexcel Imaging [64]. For the labeled dataset, NEHRI’s DesignSafe website was utilized to obtain building coordinates and the manually inspected damage class by Kijewaski-Correa et al. [65]. The Google Maps Geocoding API was then employed to get the building footprint as a polygon. Finally, the Vexcel imaging API was used to extract the corresponding image and associate it with its respective damage class. These images are extracted using the input of the time of the event and a building polygon. Following this procedure, 1072 labeled images and 16,800 unlabeled images were extracted. Figure 2 presents a sample of each class from the extracted dataset.

#### 2.1.2. Satellite Image Data

The satellite images dataset used in this study was adopted from Khajwal et al. [30], made publicly available on DesignSafe [62]. The dataset consists of 500 labeled images (examples in Figure 2) extracted from Google satellite images [63]. There are several other satellite datasets are available as open source, as discussed in the introduction section, such as the xBD dataset [71]. However, we decided not to utilize this data because it classifies damage states into four different classes (no damage, minor damage, major damage, and destroyed), which deviates from the proposed 5-class scale.

### 2.2. Deep Learning Architecture

We evaluate the performance of transformers against convolutional neural networks (CNNs), which are commonly employed for classification tasks [9,10,30,53,72]. Transformers, known for their attention mechanisms, are being increasingly adopted due to their superior performance in various deep learning tasks [60,73]. We trained two transformer models: a supervised model and a semi-supervised model. All the models are trained on Nvidia RTX 3090 with 24 GB memory. The network architectures for these models are now described.

#### 2.2.1. Supervised: Vision Transformer (ViT)

**Figure 3:**
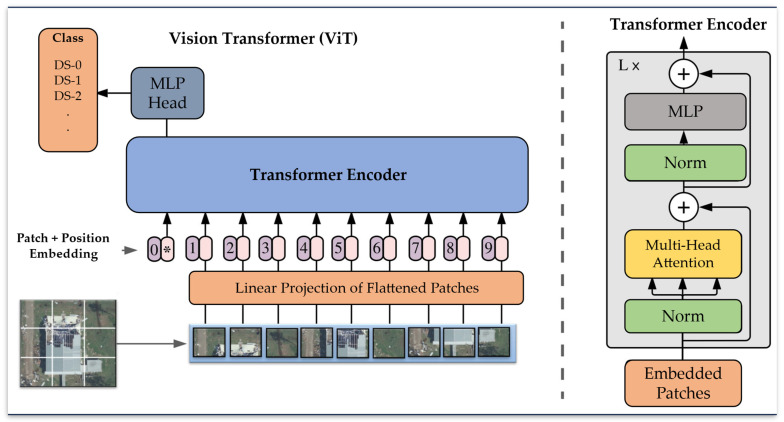
Vision Transformer (ViT) Model Architecture.

The vision transformer, also known as ViT, utilizes a transformer-based architecture to classify images [74]. It operates by dividing an image into fixed-size non-overlapping patches, followed by a linear projection of each patch. Position embeddings are then added to each patch, and the resultant sequence of vectors is passed through a standard transformer encoder [74]. The transformer encoder includes a multi-head self-attention layer, a multi-layer perceptron (MLP) layer with a gaussian error linear unit. Layer normalization is applied to each of these layers. Figure 3 visually illustrates the ViT model and its components. The hyperparameters for the training are summarized below in Table 2. The hypermeters used in the original ViT paper were directly adopted from [75]. In our study, we used a pre-trained model trained on ImageNet [76] to speed up training, improve performance, and leverage learned representations.

#### 2.2.2. Semi Supervised: Semi-ViT

The semi-supervised vision transformer (Semi-ViT) [75] is also a transformer-based model as the name suggests but utilizes unlabeled data along with labeled data. The semi-supervised learning pipeline comprises three stages: pre-training (transfer learning [35,77]), followed by supervised fine-tuning, and eventually semi-supervised fine-tuning.

**Figure 4:**
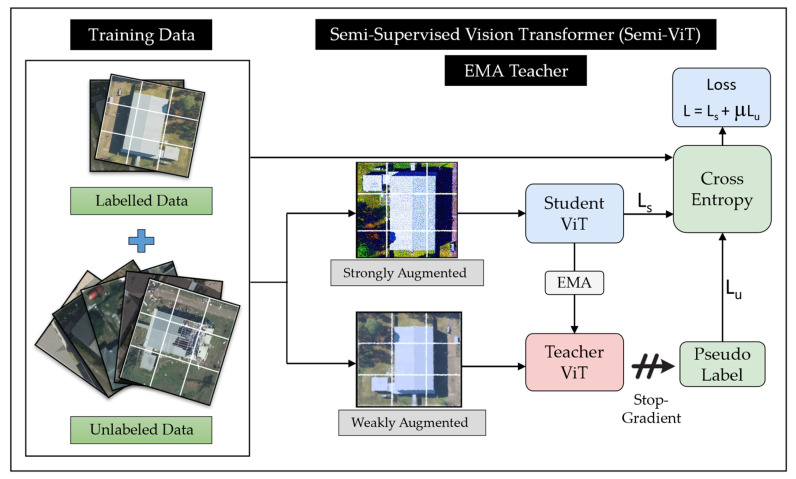
Semi-Supervised Vision Transformer (Semi-ViT): EMA Training.

In our study, we used the same pretrained model and supervised training procedure as described in the previous section. During the semi-supervised fine-tuning phase, the exponential moving average (EMA)-Teacher framework is adopted. This choice was driven by the fact that recent results from Cai et al. [75] suggest that the EMA-Teacher framework (Figure 4) provides better stability and achieves higher accuracy for semi-supervised vision transformers for classification tasks compared to the more commonly used FixMatch method [78]. The EMA-teacher framework consists of two parallel networks, the student network and the teacher network, both of which are initialized as the fully supervised ViT model is trained on labeled data.

As illustrated in Figure 4, the EMA-Teacher framework uses both labeled and unlabeled samples during training to update the weights of the student and teacher networks. Unlabeled samples undergo two types of augmentations, weak augmentations that pass through the teacher network, and strong augmentations that pass through the student network. The weak augmentations include random resized crop, random horizontal flip, and color jitter, and the strong augmentations are random resized crop, random horizontal flip, random augment [79], and random erasing [80]. When the confidence of the prediction of a weakly augmented image passed through the teacher network is above a threshold, then a pseudo-label is assigned to that image. The weights of the student network are then updated using combination batches of labeled data yielding a cross-entropy loss ($L_{s}$), and unlabeled data using a pseudo-label with a cross entropy loss $L_{u}$. The overall loss is computed as $L=L_{s}+{µL}_{u}$, where µ is the trade-off weight. The teacher network weights are then updated using the EMA method [75].

## 3. Experiments

Understanding the role and potential advantage of using the unlabeled data, selecting an optimal model architecture, and the effect of different data types are crucial considerations in the development of an automatic PDA framework. In this study, we aim to investigate three key research questions: (i) we explore the impact of incorporating unlabeled data on model prediction accuracy, with the hypothesis that augmenting labeled data with unlabeled data will improve the performance of our models, (ii) we compare the effectiveness of CNN and transformer model architectures, aiming to identify the architecture that yields superior predictive capabilities for predicting damage class, and (iii) we conduct a comparative analysis of satellite and UHRA image data types to contrast their feature extraction and generalization capabilities. In each of our models, we utilize 85% of the data for training, and 15% of the images for the testing set. All individual experiments performed are summarized in Table 3. The results of this study will contribute to the development of more accurate and robust models in the field of post-disaster damage assessment. In the following sub-section, we will outline the experiments designed to test our hypotheses.

### 3.1. Semi-Supervised Learning with Unlabeled Data

The lack of availability of labeled data presents challenges in terms of annotation, while building an unlabeled dataset is far more feasible and convenient. We aim to assess the performance of a model when labeled data is limited and investigate the extent to which incorporating unlabeled data can enhance the predictive capabilities of a model. Towards this objective, we designed two experimental cases. In the first case (UHR-Semi-100), we maintained the labeled data at 100% of the training data and utilized 100% unlabeled data. In the second case, (UHR-Semi-25) we reduced the labeled data to 25% of the training data while keeping the unlabeled data at 100% in Table 3. These cases are then compared with their corresponding supervised baseline (UHR-ViT-100 and UHR-ViT-25, respectively). By implementing these cases, we aimed to simulate real-world scenarios where the limitation in data acquisition typically affects the availability of labeled data rather than unlabeled data.

### 3.2. Comparison of CNN and Transformer Model Architectures

We performed a comparative analysis between CNN and transformer models to determine the more effective architecture for our task. To ensure a fair comparison, we kept the training and testing data consistent for both models. For this experiment, we trained a vision transformer (ViT) model (Sat-ViT-100) and compared the performance to results from a CNN model reported in Khajwal et al. [30] (Sat-CNN-100), as listed in Table 3.

### 3.3. Comparison of Satellite and UHRA Image Data Types

The objective of this experiment is to gain a quantitative and qualitative comparison between models trained on both data sources (Satellite and UHRA Images) and their adequacy for damage classification. Towards this objective, we trained two supervised ViT models on images from each data source. To ensure an unbiased experiment, we selected the buildings that were present in both the datasets. In total, there were 267 buildings common to both the satellite and UHRA datasets. These models were then tested on the test data from the same and other sources as listed in Table 4.

The naming convention for the model is as follows: {ViT}-{Training Data}-{Testing Data}. For example, ‘ViT-UHR-Sat’ represents a ViT model that was trained on UHRA images and tested on satellite images.

### 3.4. Classification Metrics

To quantitatively assess the experiment results, we employed several standard classification metrics, including accuracy, precision, recall, F1 score, and average area under the ROC (receiver operating characteristic) curve, referred to AUC-ROC in this study. Accuracy reflects the percentage of correct predictions made by the model, providing an overall measure of its correctness. Precision measures the model’s ability to correctly identify positive instances, offering insights into how well it avoids false positives. Recall evaluates the model’s ability to detect all positive cases, indicating its sensitivity to identifying actual positive instances. The F1 score, which combines precision and recall, serves as a balanced metric for accuracy, particularly in datasets with imbalanced class distribution, where certain classes may be underrepresented. Lastly, the average AUC-ROC assesses the model’s discriminative capabilities between classes. The AUC-ROC curve plots the true positive rate against the false positive rate for different classification thresholds. A higher AUC-ROC value indicates better performance in distinguishing between positive and negative classes, enhancing the model’s predictive capabilities. Together, these metrics provide a comprehensive and nuanced evaluation of the model’s performance in accurately assessing building damage classes, guiding our analysis and discussions in the subsequent sections. Refer to Table 5 for summarized evaluation metrics.

## 4. Results and Discussion

This section presents the results of the three experiments described in the previous section. These findings, summarized in Table 6, offer insights into improving model performance and data selection for an automatic PDA framework.

### 4.1. Semi-Supervised Learning with Unlabeled Data

**Figure 5:**
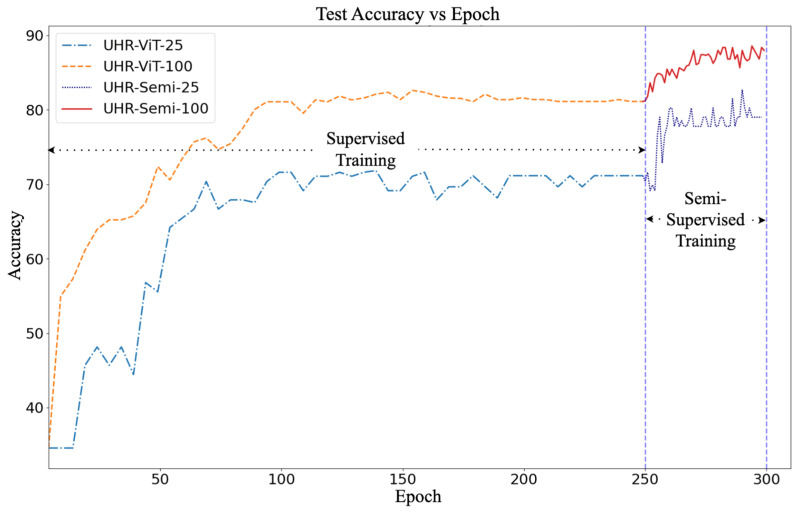
Learning Curve for Experiments UHR-Semi-25 and UHR-Semi-100.

In this section, we explore the utility of unlabeled data by conducting four experiments, denoted as UHR-ViT-100, UHR-Semi-100, UHR-ViT-25, and UHR-Semi-25 (see Table 3). The results of these experiments are depicted as two curves in Figure 5. The first part of each curve represents supervised training, and the second part represents semi-supervised training. For the supervised models, we conducted the training for 250 epochs, and for the semi-supervised model, we extended the training by an additional 50 epochs until the curve converged. The maximum accuracies achieved for UHR-ViT-100 and UHR-ViT-25 were 81% and 71%, respectively, also indicated in Table 6. Subsequently, we employed the semi-supervised approach to incorporate the unlabeled data into the training process. This led to a notable increase in accuracy of 7% and 10% for UHR-ViT-100 and UHR-ViT-25, respectively. Another notable observation was that with just 25% labeled images, the semi-supervised model was able to reach the accuracy of the supervised model with 100% labeled images. These results clearly demonstrate the effectiveness of the semi-supervised training method in enhancing the model’s performance by leveraging the additional unlabeled data.

**Figure 6:**
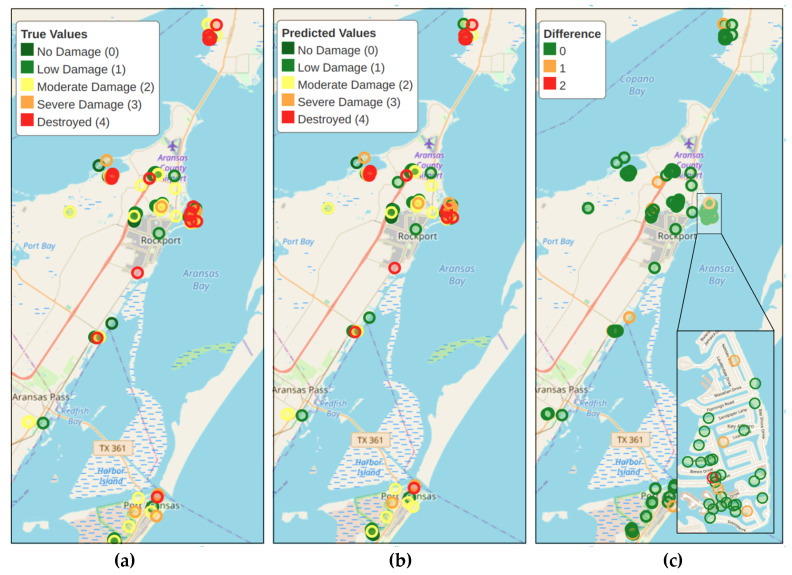
Evaluation Maps for (**a**) Ground Truth, (**b**) Predicted Values, and (**c**) Difference.

The evaluation maps (Figure 6) depict the true values, predicted class, and absolute difference between the real and predicted damage state for each building. Based on the evaluation maps, we observed that most buildings are accurately classified, with around 9% of instances showing misclassifications of ±1 class and even fewer falling into the ±2 class range (3%). Notably, there are no predictions with a difference of 3 classes, indicating that the model rarely exhibits significant errors in damage state assessment. From a practical standpoint, plotting the maps of predicted classes offers valuable insights and aids in identifying priority regions that are most affected after a disaster.

### 4.2. Comparison of CNN and Transformer Model Architectures

In this section, we present a comparison between a CNN and transformer model. The primary aim is to determine which model architecture is more effective for the building damage classification. The transformer-based model displayed a remarkable 18% higher accuracy compared to the CNN-based model (see Sat-CNN-100 and Sat-ViT-100 in Table 6). This improvement was consistent across other performance metrics as well, including precision, recall, and F1 score. In experiment Sat-CNN-100, the model achieved an accuracy of 55%, and an average F1 score of 54% [30]. In contrast, Sat-ViT-100 yielded significantly improved results with an accuracy of 73%, and an average F1 score of 72%. An essential observation here is that the model surpasses human-level accuracy on satellite images, achieving a 3% improvement over the reported 70% human accuracy [54,81]. This result establishes the model’s reliability and suitability for practical applications.

**Figure 7:**
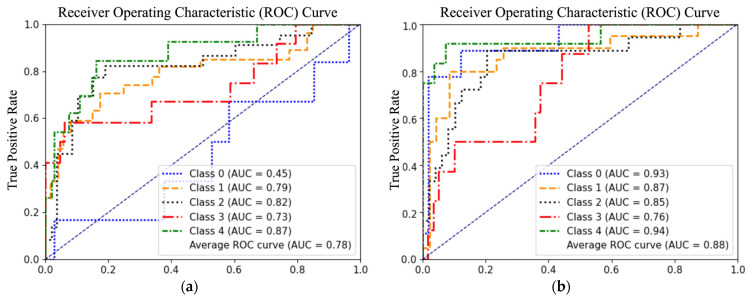
ROC Curve for (**a**) Sat-CNN-100 and (**b**) Sat-ViT-100.

To gain further insights into the predictive capabilities of the models, we compared the ROC curves shown in Figure 7. The ROC curve analysis showed a higher AUC-ROC for the transformer model, indicating its superior ability to discriminate between classes effectively for all the classes. Another observation in both results is the lower AUC-ROC value for class 3, indicating the maximum uncertainty in prediction. This uncertainty is expected for satellite images, and it also aligns with observations from a study on human assessments [81]. Lastly, comparing damage class 0 in both cases, the CNN model exhibits poor predictive capabilities, performing close to a random classifier, as evidenced by its ROC falling below 0.5. Conversely, the transformer model demonstrates higher discriminative capability, with an AUC-ROC of 0.93.

The overall results indicate that the transformer-based architecture has better ability to learn high-level features and capture complex patterns. This might be due to the transformer’s attention mechanisms, which appear to be advantageous for handling spatial features in satellite images. Spatial features refer to the specific characteristics and patterns within an image. Vision transformers perform better than CNNs in terms of extracting spatial features due to their ability to preserve the spatial information of the embedded patches and capture long-range dependencies between image regions [82,83]. In the context of damaged buildings, the key distinguishing areas are the damaged and undamaged sections.

### 4.3. Comparison of Satellite and UHRA Image Data Types

The following section presents the comparison between satellite and UHRA data sources. The results of all the experiments are summarized in Table 7. According to the experimental results, the model trained on UHRA images and tested on a satellite images yielded an accuracy of 58%. Through our series of experiments, we can draw two conclusions that suggest UHRA images are more suitable for training the ViT model.

We notice that the models trained on UHRA images demonstrate better generalizability capabilities when tested on satellite images. The ViT-UHR-Sat model achieved an accuracy and F1 score of 58% and 62%, respectively. The ViT-Sat-UHR model achieved a lower accuracy of 41% and F1 score of 39%. This indicates that the model effectively learned features from the UHRA images and was able to generalize the satellite data well compared to the model trained on satellite images to perform generalization on UHR images.

**Figure 8:**
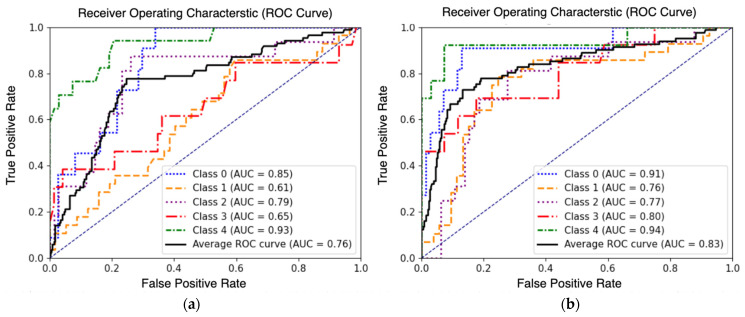
ROC Curve Analysis of (**a**) ViT-Sat-UHR (**b**) ViT-UHR-Sat.

**Figure 9:**
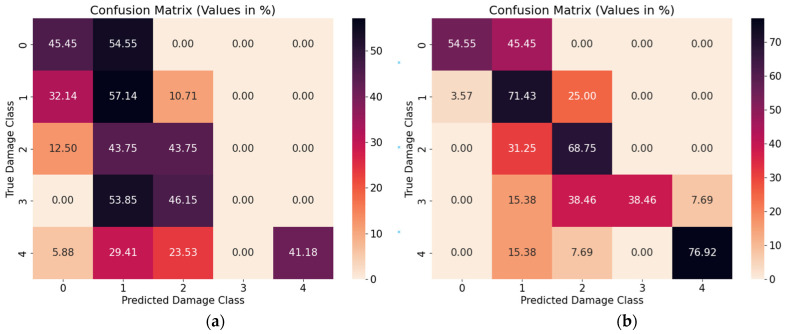
Confusion Matrices for (**a**) ViT-Sat-UHR and (**b**) ViT-UHR-Sat.

Secondly, the AUC-ROC curve (Figure 8) reveals that the ViT-UHR-Sat exhibits superior discriminative capabilities in distinguishing between different classes. The average AUC-ROC scores achieved by ViT-UHR-Sat and ViT-Sat-UHR are 83% and 76%, respectively, reinforcing the higher discriminative capabilities of ViT-UHR. Moreover, ViT-UHR-Sat successfully overcomes the challenges associated with classifying damage state 3 when trained on satellite images, as discussed in the previous section (see Figure 7a and Figure 8b). The confusion matrix in Figure 9 highlights this observation as well; the ViT-Sat-UHR model struggles to accurately predict the intermediate damage classes (DS-1, DS-2 and DS-3). Another observed issue is the misclassification of damage state 3 as damage states 2 and 1. In contrast, the ViT-UHR-Sat model demonstrates better performance comparatively.

We also study the resolution and accuracy of the class activation mappings or CAMs produced by networks trained on these datasets. A CAM [84] can identify specific regions in an image that a model is focusing on while making a classification decision. In this study, we are using Eigen-CAM, proposed by Muhammad et al. [85]. We perform the CAM on the layers before the final activation block to avoid the zero-gradient problem in transformer models [86].

**Figure 10:**
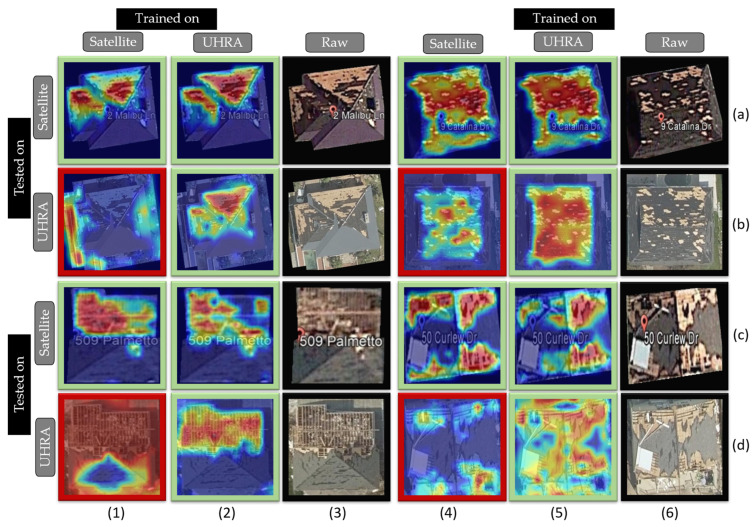
Class activation maps (CAMs) identifying good CAMs in green boxes and bad CAMs in red boxes.

Figure 10 presents the CAMs for an individual building across various experimental settings where the model is trained and tested on different combinations of data sources. The CAMs highlighted in green boxes are considered accurate, while those in red boxes are deemed less reliable. Upon examining the CAMs, it becomes apparent that the models are striving to differentiate between regions of damaged and undamaged rooftops. From the CAM analysis, two noteworthy observations can be made: (i) The CAMs for the model trained and tested on UHRA images are quite accurate and precise in detecting damaged regions (2-b, 5-b, 2-d, and 5-d). A similar performance is observed when the model is trained and tested on satellite images (1-a, 4-a, 1-c, and 4-c). (ii) The models trained on UHRA and tested on satellite images (ViT-UHR-Sat), produce good CAMs (2-a, 5-a, 2-c, and 5-c) and effectively identify damaged regions. However, the model trained on satellite images (ViT-Sat-UHR) does not perform well (1-b, 4-b, 1-d and 1-d) when tested on UHRA images. Conversely, the ViT-UHR-Sat model successfully distinguishes between buildings and the background, yielding accurate CAMs.

The results from CAMs, ROC curve, and the confusion matrix affirm that the model trained on UHRA images demonstrates better generalizability and discriminative capabilities among all classes. This reinforces the practical value of UHRA images in enhancing the framework’s performance for accurate building damage assessment.

### 4.4. Limitations

This study presents novel insights into building damage assessment using satellite and UHRA data. While the proposed framework has been extensively validated for post-hurricane damage assessments, and could potentially be extended to other related scenarios as well, the following limitations are acknowledged:

1. Above-Ground Structures Only: The methodology is tailored for above-ground structures and would not be suitable for subsurface assessment.
2. Cloud Cover Impact: The flight altitude for capturing UHRA images is approximately 2 km, making clouds below this altitude a potentially significant limitation in the damage detection process.
3. Roof Damage Sensitivity: While the sensitivity to roof damage serves as a valuable indicator for the PDA, it may not be equally informative for evaluating damage caused by other disasters where roof damage is not a good indicator of overall structural health.

## 5. Conclusions

This paper addressed key challenges in building an efficient, accurate, and automatic preliminary disaster assessment (PDA) framework. The novel contributions of our research stemmed from the adoption of (i) UHRA images, (ii) unlabeled data, and (iii) vision-transformer models. We investigated the impact of leveraging unlabeled data to improve classification accuracy, compared CNN and transformer model architectures, and quantitatively assessed the usefulness of satellite and ultra-high-resolution aerial (UHRA) images. The results demonstrated that the semi-supervised model with UHRA images is able to attain a state-of-the-art 5-class accuracy of 88%, yielding a 33% improvement over the previous state-of-the-art CNN trained on satellite data. Our experiments also demonstrated the efficacy of unlabeled data in improving the accuracy of the supervised model (UHRA-ViT-100) by 7%. A comparison of baseline supervised architectures on satellite data only, demonstrated the transformer’s ability to learn high-level features and achieve an overall accuracy of 73% vs. 55% for the CNN model. Furthermore, incorporating UHRA images for training not only enhances the model’s ability to generalize to different datasets but also improves its performance in distinguishing between classes. The results were verified by analyzing class activation maps (CAMs) to better interpret the models. The results from this study will significantly accelerate and improve post-disaster assessment and the overall recovery process. The proposed framework offers increased speed and accuracy compared to current automated PDA frameworks. Adoption of the framework can prove valuable across various phases of disaster recovery, such as expediting the identification of priority regions for detailed inspection, streamlining the processing of a large number of federal financial aid applications post-disaster, and facilitating the cost estimation for recovery.

## Figures and Tables

**Table 1:** Scale mapping of damage scale.

Proposed Scale	Individual Assessment (IA)	HAZUS	NEHRI Design Safe [68]	Description *			
				Roof Cover Damage	Sliding Damage	Door and Windows Failure	Roof Sheathing Failures
0	NA	No Damage	0	≤10%	≤1 Panel	None	None
1	Affected	Minor	1	>10% to ≤25%	>1% to ≤25%	1 or 2	None
2	Minor	Moderate	2	>25%	>25%	>2% to ≤50%	>0% to ≤25%
3	Major	Severe	3	>25%	>25%	>50%	>25% with minor connection failure
4	Destroyed	Destroyed	4	>25%	>25%	>50%	>25% with major connection failure
NA	Inaccessible	NA	NA	Damage to residence cannot be visually verified			

* Complete description of each damage class is discussed in [4,67].

**Table 2:** Model Hyperparameters.

Hyperparameter	ViT	Semi-ViT
Optimizer	AdamW	AdamW
Base Learning Rate	0.001	0.0025
Weight Decay	0.05	0.05
Mixup	0.8	0.8
Cutmix	1.0	1.0
Epochs	250	50

**Table 3:** Summary of experiments.

Experiment Name	Data Type	Model Architecture	Labeled (%)	Unlabeled (%)	Deep Learning Method
Sat-CNN-100	Satellite	CNN	100	0	Supervised
Sat-ViT-100	Satellite	Transformer (ViT)	100	0	Supervised
UHR-ViT-100	UHRA	Transformer (ViT)	100	0	Supervised
UHR-ViT-25	UHRA	Transformer (ViT)	25	0	Supervised
UHR-Semi-100	UHRA	Transformer (Semi-ViT)	100	100	Semi-Supervised
UHR-Semi-25	UHRA	Transformer (Semi-ViT)	25	100	Semi-Supervised

**Table 4:** Summary of inter-data experiments.

Model Name	Trained on	Tested on
ViT-UHR-UHR	UHRA (213)	UHRA (54)
ViT-UHR-Sat	UHRA (213)	Satellite (54)
ViT-Sat-Sat	Satellite (213)	Satellite (54)
ViT-Sat-UHR	Satellite (213)	UHRA (54)

Values in the bracket () represent no. of images.

**Table 5:** Summary of evaluation metrics.

Metric	Formula
Accuracy	(True Positives + True Negatives)/Total
Precision	True Positives/(True Positives + False Positives)
Recall	True Positives/(True Positives + False Negatives)
F1 Score	2 × (Precision × Recall)/(Precision + Recall)
Average AUC-ROC	Average of Area Under the ROC Curve for all Classes

**Table 6:** Performance report for different experiments.

Experiment #	Accuracy (%)	Average F1 (%)	Precision (%)	Recall (%)	Average AUC-ROC (%)
UHR-Semi-100	88	88	89	88	96
UHR-ViT-100	81	77	79	77	94
UHR-Semi-25	81	83	84	82	91
Sat-ViT-100	73	72	72	73	88
UHR-ViT-25	71	68	70	68	91
Sat-CNN-100	55	54	55	55	78

**Table 7:** Performance report on inter-dataset testing.

Model Name	Accuracy (%)	F1 (%)	Average AUC-ROC (%)
ViT-UHR-Sat	58	62	83
ViT-Sat-UHR	41	39	76
ViT-UHR-UHR	71	68	91
ViT-Sat-Sat	67	66	91

## Data Availability

The satellite data can be accessed online at https://www.designsafe-ci.org/data/browser/public/designsafe.storage.published/PRJ-3692 (accessed on 4 August 2023). The Vexcel Image data is not publicly accessible.
